# Characterization of the Asymmetry of the Cardiac and Sympathetic Arms of the Baroreflex From Spontaneous Variability During Incremental Head-Up Tilt

**DOI:** 10.3389/fphys.2019.00342

**Published:** 2019-04-02

**Authors:** Beatrice De Maria, Vlasta Bari, Beatrice Cairo, Emanuele Vaini, Murray Esler, Elisabeth Lambert, Mathias Baumert, Sergio Cerutti, Laura Dalla Vecchia, Alberto Porta

**Affiliations:** ^1^IRCCS Istituti Clinici Scientifici Maugeri, Milan, Italy; ^2^Department of Cardiothoracic, Vascular Anesthesia and Intensive Care, IRCCS Policlinico San Donato, Milan, Italy; ^3^Department of Biomedical Sciences for Health, University of Milan, Milan, Italy; ^4^Human Neurotransmitters Laboratory, Baker IDI Heart and Diabetes Institute, Melbourne, VIC, Australia; ^5^Faculty of Health, Arts and Design, Iverson Health Innovation Research Institute, Swinburne University of Technology, Hawthorn, VIC, Australia; ^6^School of Electrical and Electronic Engineering, The University of Adelaide, Adelaide, SA, Australia; ^7^Department of Electronics Information and Bioengineering, Politecnico di Milano, Milan, Italy

**Keywords:** hysteresis, muscle sympathetic nerve activity, MSNA, baroreflex sequence analysis, phase-rectified signal averaging, heart rate variability, autonomic nervous system, cardiovascular control

## Abstract

Hysteresis of the baroreflex (BR) is the result of the different BR sensitivity (BRS) when arterial pressure (AP) rises or falls. This phenomenon has been poorly studied and almost exclusively examined by applying pharmacological challenges and static approaches disregarding causal relations. This study inspects the asymmetry of the cardiac BR (cBR) and vascular sympathetic BR (sBR) in physiological closed loop conditions from spontaneous fluctuations of physiological variables, namely heart period (HP) and systolic AP (SAP) leading to the estimation of cardiac BRS (cBRS) and muscle sympathetic nerve activity (MSNA) and diastolic AP (DAP) leading to the estimation of vascular sympathetic BRS (sBRS). The assessment was carried out in 12 young healthy subjects undergoing incremental head-up tilt with table inclination gradually increased from 0 to 60°. Two analytical methods were exploited and compared, namely the sequence (SEQ) and phase-rectified signal averaging (PRSA) methods. SEQ analysis is based on the detection of joint causal schemes representing the HP and MSNA burst rate delayed responses to spontaneous SAP and DAP modifications, respectively. PRSA analysis averages HP and MSNA burst rate patterns after aligning them according to the direction of SAP and DAP changes, respectively. Since cBRSs were similar when SAP went up or down, hysteresis of cBR was not detected. Conversely, hysteresis of sBR was evident with sBRS more negative when DAP was falling than rising. sBR hysteresis was no longer visible during sympathetic activation induced by the orthostatic challenge. These results were obtained via the SEQ method, while the PRSA technique appeared to be less powerful in describing the BR asymmetry due to the strong association between BRS estimates computed over positive and negative AP variations. This study suggests that cBR and sBR provide different information about the BR control, sBR exhibits more relevant non-linear features that are evident even during physiological changes of AP, and the SEQ method can be fruitfully exploited to characterize the BR hysteresis with promising applications to BR branches different from cBR and sBR.

## Introduction

The baroreflex (BR) can be seen as a composite reflex formed by several arms simultaneously adjusting multiple physiological variables in response to the same arterial pressure (AP) variation. Among these branches the cardiac BR (cBR) and the vascular sympathetic BR (sBR) react to AP changes, respectively, with parallel variations of heart period (HP) (Smyth et al., 1969; Pickering et al., 1972) and antiparallel variations of sympathetic nerve activity. Sympathetic traffic is commonly surrogated in humans with muscle sympathetic nerve activity (MSNA) recorded via microneurographic technique (Sundlof and Wallin, 1978). The characterization of BR is usually based on the estimation of the BR sensitivity (BRS) representing the variation of the target variable, such as HP or MSNA, per unit change of AP. Cardiac BRS (cBRS) is mostly estimated by observing the variation of HP in response to a unit modification of systolic AP (SAP) (Smyth et al., 1969; Pickering et al., 1972), while vascular sympathetic BRS (sBRS) is more frequently assessed by measuring the variation of MSNA, or probability of occurrence of the MSNA burst, per unit change of diastolic AP (DAP) (Sundlof and Wallin, 1978; Kienbaum et al., 2001). The cBRS is positive because HP decreases in response to a SAP fall and HP increases in reaction to a SAP rise. Conversely, sBRS is negative given that the amplitude (or area) and the likelihood of the occurrence of the MSNA burst increase in response to DAP drops and they rise when DAP decreases.

Pharmacological studies in young healthy individuals suggested that the cBR and sBR arms exhibit an asymmetric behavior with different BRS computed over positive and negative AP changes (Pickering et al., 1972; Sundlof and Wallin, 1978; Studinger et al., 2007; Studinger et al., 2009). This asymmetry leads to the phenomenon of hysteresis with distinct trajectories covered by the set point in the planes (SAP,HP) and (DAP,MSNA) when AP rises and falls (Rudas et al., 1999; Studinger et al., 2007, 2009; Hart et al., 2011). More specifically, the cBR responds to the same absolute variation of SAP with a larger absolute variation of HP during SAP rise than fall (Pickering et al., 1972; Rudas et al., 1999). Conversely, the sBR reacts to the same absolute variation of DAP with a larger probability of observing an MSNA burst during DAP decrease than increase. These results are evident when important variations of AP are imposed via the administration of vasoactive drugs (Pickering et al., 1972; Sundlof and Wallin, 1978; Rudas et al., 1999; Studinger et al., 2007, 2009), but it is unclear whether they can be confirmed in presence of spontaneous, and likely small, AP changes. Indeed, the studies that computed BRS by separating positive and negative AP variations provided an incomplete answer given that the issue of testing BR hysteresis was not straightly tackled (Parati et al., 1988; De Maria et al., 2018), or the analysis was limited to a single arm of the BR (Martin-Vazquez and Reyes Del Paso, 2010; Davydov et al., 2018) or a static approach was exploited with limited possibility to explore causal relations (Hart et al., 2011). This lack limits the comprehension of the BR functioning and its arms in physiological conditions (Taylor et al., 2015; Marchi et al., 2016b).

Therefore, the aim of this study is to perform the simultaneous characterization of cBR hysteresis from spontaneous variability of HP and SAP and sBR hysteresis from spontaneous fluctuations of MSNA burst rate and DAP during incremental head-up tilt in young healthy individuals (Lambert et al., 2008). cBRS and sBRS are computed via the sequence (SEQ) method (Bertinieri et al., 1985) and via the phase-rectified signal averaging (PRSA) (Bauer et al., 2010; Muller et al., 2012). The SEQ technique extracts joint causal parallel HP-SAP ramps (Parati et al., 1988) and joint causal antiparallel MSNA-DAP ramps (Marchi et al., 2016b), while PRSA aligns HP and MSNA burst rate patterns according to the sign of SAP and DAP changes, respectively. The assessment of cBRS and sBRS is carried out by separately considering positive and negative AP variations (De Maria et al., 2018). The simultaneous application of both SEQ and PRSA methods allows us to compare the ability of the two approaches in typifying the BR hysteresis, while the simultaneous description of cBR and sBR hystereses allows us to stress peculiarity of different BR arms in physiological conditions.

## Materials and Methods

### Experimental Protocol

Twelve young healthy subjects (9 females; age from 20 to 36 years, median = 22.5 years; body mass index from 18.6 to 28.4 kg⋅m^-2^, median = 24.2 kg⋅m^-2^) were enrolled in the study. The experimental protocol was fully described in Lambert et al. (2008). Briefly, incremental graded head-up tilt test, starting from 0 to 60°, was performed. Subjects were consecutively tilted at 0, 20, 30, 40, and 60° (T0, T20, T30, T40, and T60, respectively) and maintained in each position for 10 min. The subjects never returned to the supine position and tilt table inclination was incremented from the previous one. The test was performed in the morning, 1 h after a light breakfast and after at least a 12-h caffeine free period. The subjects breathed spontaneously but they were not allowed to talk. The experimental protocol was approved by the Alfred Hospital Ethics Review Committee (no. 144/06) and conformed to the relevant guidelines of the National Health and Medical Research Council of Australia and to the principles of the Declaration of Helsinki. All subjects signed written informed consent before the test.

Electrocardiogram (ECG), invasive AP, and MSNA signals were recorded for the overall duration of the test. ECG was monitored using a single III lead amplifier (ADInstruments, Castle Hill, NSW, Australia). AP signal was obtained by cannulating percutaneously the radial artery (3F, 5 cm, Cook catheter). A clinical microneurography (IOWA Nerve Traffic Analyzer, model 662C-3, Department of Bioengineering, The University of Iowa, Iowa, IA, United States) was used to record the multiunit sympathetic nerve discharges in postganglionic fibers distributed to the skeletal muscle vasculature. A tungsten microelectrode (FHC, Bowdoinham, Maine, United States) was percutaneously inserted in the peroneal nerve and adjusted in order to obtain a satisfactory MSNA signal (Lambert et al., 2008). The raw MSNA signal was band-pass filtered (700–2000 Hz), amplified, rectified and integrated (time constant of 0.1 s). The integrated MSNA signal was utilized for further analysis. The sampling rate of the recorded signals (i.e., ECG, AP and integrated MSNA) was 1000 Hz (PowerLab system, model ML785/8SP, ADInstruments, Castle Hill, NSW, Australia). Out of all 12 subjects, the recordings of one subject during T30 and T40 were excluded for poor quality, while 5 subjects did not complete the experimental protocol during T60.

### Beat-to-Beat Variability Series Extraction

The *k*th HP, *HP*(*k*), where *k* is the cardiac beat counter, was calculated as the temporal distance between two consecutive R-wave peaks detected on the ECG signal. QRS complexes were identified when the absolute first derivative of the ECG overcame a predefined threshold. R-wave peaks were fixed by means of parabolic interpolation. The minimum and maximum values of the AP signal within *HP*(*k*) were considered as the *k*th DAP, *DAP*(*k*), and the *k*th SAP, *SAP*(*k*), respectively. *DAP*(*k*) preceded in time *SAP*(*k*). All the identified fiduciary points (i.e., R peaks, SAP and DAP values) were visually checked and manually corrected in case of erroneous or missed detections. In presence of ectopic beats, corrections were performed over the series via cubic spline interpolation taking as onset and offset the closest values unaffected by the ectopies. No more than 5% of correction was allowed.

From the integrated MSNA signal the variability of the MSNA burst rate was obtained as described in Marchi et al. (2016a). The first step was the detection of the MSNA bursts over the entire recording. To account for the latency of the sBR the MSNA bursts were searched in a temporal window ranging from 0.9 to 1.7 s from the R-wave peak (Sundlof and Wallin, 1978; Kienbaum et al., 2001; Diedrich et al., 2009). A running threshold calculated as a fraction of the maximum burst amplitude in the overall signal and updated on a beat-to-beat basis, allowed the detection of the MSNA bursts by overcoming the problems related to bursts amplitude variation and baseline wandering (Diedrich et al., 2009). The second step to obtain the MSNA burst rate variability series was the counting of the previously detected MSNA bursts in a moving time window of 5 s that was advanced in steps of 1 ms. The obtained step-wise burst-count MSNA signal was filtered with a cut-off frequency equal to 0.5 Hz, thus focusing the typical range of frequencies of spontaneous variability in humans (Task Force of the European Society of Cardiology the North American Society of Pacing Electrophysiology, 1996) and the filtered signal was sampled at the occurrence of the first R-wave peak representing the onset of the *HP*(*k*) and the correspondent value was indicated as *MSNA*(*k*). The values of the MSNA burst rate variability series were divided by the frame length (i.e., 5 s), thus representing the number of bursts occurring in 1 s and its units are bursts⋅s^-1^. Analyses were carried out over the beat-to-beat series HP = {*HP*(*k*), *k* = 1,…,*N*}, SAP = {*SAP*(*k*), *k* = 1,…,*N*}, DAP = {*DAP*(*k*), *k* = 1,…,*N*}, and MSNA = {*MSNA*(*k*), *k* = 1,…,*N*}, where *N* = 300 cardiac beats according to the typical sequence length exploited in short-term analysis of cardiovascular control (Task Force of the European Society of Cardiology the North American Society of Pacing Electrophysiology, 1996).

### cBRS Estimation via the SEQ Method

cBRS was computed according to the SEQ method (Bertinieri et al., 1985) as implemented in Porta et al. (2000). More specifically, the SEQ method for the cBR analysis is based on the search of ordered HP and SAP sequences ***HP***(*k* + *τ_cBR_*) = [*HP*(*k* + *τ_cBR_*), *HP*(*k* + *τ_cBR_* - 1), *HP*(*k* + *τ_cBR_* - 2), *HP*(*k* + *τ_cBR_* - 3)] and ***SAP***(*k*) = [*SAP*(*k*), *SAP*(*k* - 1), *SAP*(*k* - 2), *SAP*(*k* - 3)] formed by four consecutive HP and SAP values corresponding to three HP and SAP variations defined as Δ*HP*(*k* + *τ_cBR_*) = *HP*(*k* + *τ_cBR_*) -*HP*(*k* + *τ_cBR_* - 1) and Δ*SAP*(*k*) = *SAP*(*k*) -*SAP*(*k* - 1). The sequence ***SAP***(*k*) precedes ***HP***(*k* + *τ_cBR_*), where *τ_cBR_* represents the cBR latency expressed in cardiac beats. If ***HP***(*k* + *τ_cBR_*) and ***SAP***(*k*) sequences feature all positive variations, they were referred to as SEQ+. Therefore, SEQ+ is a joint HP-SAP scheme formed by positive HP and SAP ramps. Conversely, if ***HP***(*k* + *τ_cBR_*) and ***SAP***(*k*) sequences feature all negative variations they were termed as SEQ-. Therefore, SEQ- is a joint HP-SAP scheme formed by negative HP and SAP ramps. All SEQ+ and SEQ- joint schemes were considered of cBR origin regardless of the magnitude of total, or partial, SAP and HP variations and the strength of the linear association between HP and SAP values (Porta et al., 2013). The robustness of the results was checked by applying more usual thresholds, namely absolute total SAP variation > 1 mmHg; absolute total HP variation > 5 ms; correlation coefficient > 0.85 (Parati et al., 1988). The latency *τ_cBR_* was optimized on an individual basis according to procedure proposed in Porta et al. (2018b) in the range from 0 to 4 beats according to the rapidity of the vagal arm of the cBR acting within the next cardiac beat following the current SAP (i.e., *τ_cBR_* = 0 beats) (Eckberg, 1976; Baselli et al., 1994) and the slower actions that should be exhausted within a time interval of 3–4 s (Baskerville et al., 1979). The cBRS was separately computed over SEQ+ and SEQ- patterns. Over each type of joint pattern (i.e., SEQ+ and SEQ-), the slope of the linear regression in the plane [*SAP*(*k*), *HP*(*k* + *τ_cBR_*^o^)], where *τ_cBR_*^o^ is the optimal *τ_cBR_*, was computed and its average value over all joint HP-SAP patterns belonging to the same family (i.e., SEQ+ or SEQ-) was taken as an estimate of the cBRS. cBRS was labeled as cBRS_SEQ+_ and cBRS_SEQ-_ according to the type of joint HP-SAP pattern. Both cBRS_SEQ+_ and cBRS_SEQ-_ were non-negative and expressed in ms⋅mmHg^-1^.

### sBRS Estimation via the SEQ Method

sBRS was computed according to the SEQ method proposed in Marchi et al. (2016b). More specifically, the SEQ method for the sBR analysis is based on the search of ordered MSNA burst rate and DAP sequences ***MSNA***(*k* + *τ_sBR_*) = [*MSNA*(*k* + *τ_sBR_*), *MSNA*(*k* + *τ_sBR_* - 1), *MSNA*(*k* + *τ_sBR_* - 2), *MSNA*(*k* + *τ_sBR_* - 3)] and ***DAP***(*k*) = [*DAP*(*k*), *DAP*(*k* - 1), *DAP*(*k* - 2), *DAP*(*k* - 3)] formed by four consecutive MSNA burst rate and DAP values corresponding to three MSNA burst rate and DAP variations Δ*MSNA*(*k* + *τ_sBR_*) = *MSNA*(*k* + *τ_sBR_*) -*MSNA*(*k* + *τ_sBR_* - 1) and Δ*DAP*(*k*) = *DAP*(*k*) -*DAP*(*k* - 1). The sequence ***DAP***(*k*) precedes ***MSNA***(*k* + *τ_sBR_*), where *τ_sBR_* represents the sBR latency expressed in cardiac beats. If the ***MSNA***(*k* + *τ_sBR_*) sequence features all negative variations, while the ***DAP***(*k*) one exhibits all positive variations, it is referred to as SEQ+. Therefore, SEQ+ is a joint MSNA-DAP scheme formed by negative MSNA and positive DAP ramps. Conversely, if the ***MSNA***(*k* + *τ_sBR_*) sequence features all positive variations, while the ***DAP***(*k*) one exhibits all negative variations, it is termed SEQ-. Therefore, SEQ- is a joint MSNA-DAP scheme formed by positive MSNA and negative DAP ramps. All SEQ+ and SEQ- joint schemes were considered to be of sBR origin regardless of the magnitude of total, or partial, DAP and MSNA burst rate variations and the strength of the linear association between MSNA burst rate and DAP values. The robustness of the results was checked by applying standard thresholds, namely absolute total DAP variation > 1 mmHg; absolute total MSNA burst rate variation > 0 bursts⋅s^-1^; absolute correlation coefficient > 0.85 (Marchi et al., 2016b). The latency *τ_sBR_* was optimized on an individual basis according to procedure proposed in Porta et al. (2018b) in the range from 0 to 3 beats according to the delay of sBR in acting on MSNA after sensing AP (Sundlof and Wallin, 1978; Kienbaum et al., 2001; Diedrich et al., 2009). The sBRS was separately computed over SEQ+ and SEQ- patterns by following the same regression line approach as in the cases of cBR but applied in the plane [*DAP*(*k*), *MSNA*(*k* + *τ_sBR_*^o^)], where *τ_sBR_*^o^ is the optimal *τ_sBR_*. sBRS was labeled as sBRS_SEQ+_ and sBRS_SEQ-_ according to the type of joint MSNA-DAP pattern. Both sBRS_SEQ+_ and sBRS_SEQ-_ were non-positive and expressed in bursts⋅s^-1^⋅mmHg^-1^.

### cBRS Estimation via the PRSA Method

The PRSA method for the cBRS estimation was originally described in Bauer et al. (2010) and Muller et al. (2012). Defined as the anchor time the cardiac beat index *k* where SAP increases [i.e., Δ*SAP*(*k*) > 0], a sequence of 15 consecutive HPs around the anchor time *k* + *τ_cBR_* was selected, where *τ_cBR_* is the cBR latency. Each HP sequence was composed by the seven HPs preceding *HP*(*k* + *τ_cBR_*), *HP*(*k* + *τ_cBR_*), and the seven HPs following *HP*(*k* + *τ_cBR_*). All the identified HP segments were aligned at the anchor times. Defined *X*(0) as the mean of all HPs at the anchor time, *X*(-1) as the mean of the HPs preceding the anchor time, *X*(-2) as the mean of the HPs at two beats before the anchor time, and *X*(1) as the mean of the HPs immediately following the anchor time, the PRSA estimate of cBRS driven by positive SAP variations (cBRS_PRSA+_) was calculated as cBRS_PRSA+_ = 1/4 [*X*(0) + *X*(1) -*X*(-1) - *X*(-2)]. cBRS_PRSA+_ was expressed in ms. Given that cBRS_PRSA+_ was not expressed in usual cBRS units, a normalized version of the original PRSA method (nPRSA) was devised (Muller et al., 2012). nPRSA estimate of cBRS_PRSA+_ (cBRS_nPRSA+_) was obtained by dividing cBRS_PRSA+_ by the averaged ΔSAP(k). cBRS_nPRSA+_ was expressed in ms⋅mmHg^-1^. In the original version anchor times were defined exclusively in correspondence of Δ*SAP*(*k*) > 0. In De Maria et al. (2018) it was proposed to compute cBRS_PRSA-_ and cBRS_nPRSA-_ as well by simply repeating the same procedure as before over the anchor times where Δ*SAP*(*k*) < 0. The sign of cBRS_PRSA-_ was inverted to preserve the non-negativity of cBRS estimates. cBRS_PRSA-_ was expressed in ms, while cBRS_nPRSA-_ in ms⋅mmHg^-1^. In agreement with the fastness of vagal arm of cBR *τ_cBR_* was assigned to 0 beats (Eckberg, 1976; Baselli et al., 1994).

### sBRS Estimation via the PRSA Method

In this study we applied the PRSA method (Bauer et al., 2010; Muller et al., 2012) with the extension proposed in De Maria et al. (2018) to perform sBR analysis by separating the contributions given by positive and negative DAP variations. Briefly, the procedure described in previous *Section* was repeated by substituting SAP with DAP and HP with MSNA burst rate. Markers computed via the PRSA method over positive and negative DAP variations were labeled as sBRS_PRSA+_ and sBRS_PRSA-_, respectively, and those calculated via the nPRSA technique were termed as sBRS_nPRSA+_ and sBRS_nPRSA-_, respectively. The sign of sBRS_PRSA-_ was inverted to preserve the non-positivity of sBRS estimates. sBRS_PRSA+_ and sBRS_PRSA-_ were expressed in bursts⋅s^-1^, and sBRS_nPRSA+_ and sBRS_nPRSA-_ in bursts⋅s^-1^⋅mmHg^-1^.

### Statistical Analysis

After pooling together all the data regardless of the experimental condition, the significance of the difference between cBRS, or sBRS, computed over positive and negative AP variations was tested by means of paired *t*-test, or Wilcoxon signed rank test when appropriate. If paired analysis could not be carried out because cBRS, or sBRS, could not be computed over both positive and negative AP changes, unpaired *t*-test, or Mann–Whitney rank sum test when appropriate, was applied. Two-way analysis of variance (Holm–Sidak test for multiple comparisons) was used to check the significance of the differences between cBRS, or sBRS, indexes computed separately according to the sign of the AP variation within the same experimental condition (i.e., T0, T20, T30, T40, and T60) and between-condition differences (T20, T30, T40, and T60 versus T0) within the same type of marker (i.e., calculated over positive or negative AP changes). Pearson correlation analysis was carried out to assess the significance of the association between cBRS, or sBRS, estimates and the sine of the tilt table angle (i.e., 0, 20, 30, 40, and 60°) taken as an effective marker of the magnitude of the orthostatic challenge. After pooling together all the data regardless of the experimental condition, the same tool was carried out to assess the correlation between the cBRS estimates derived from positive and negative SAP changes, and between the sBRS estimates derived from positive and negative DAP variations. Pearson product moment correlation coefficient *r* and type I error probability *p* were calculated. Statistical analysis was repeated for all the methods utilized to estimate cBRS and sBRS (i.e., SEQ, PRSA, and nPRSA techniques). A *p* < 0.05 was always deemed as significant. Statistical analysis was carried out using a commercial statistical program (Sigmaplot, Systat Software, Inc., Chicago, IL, United States, version 11.0).

## Results

cBRS and sBRS were computed in all the subjects in all the experimental conditions by PRSA and nPRSA methods (i.e., 100% of the recordings) and this performance held regardless of the sign of the AP change. Conversely, SEQ approach had more limited performance and was able to measure cBRS and sBRS, respectively, in 88 and 92% of the recordings over positive AP variations and, respectively, in 92 and 98% of the recordings over negative AP variations. The reason for this inability was the lack of SEQ+ or SEQ- patterns in some subjects in some experimental conditions.

**FIGURE 1:**
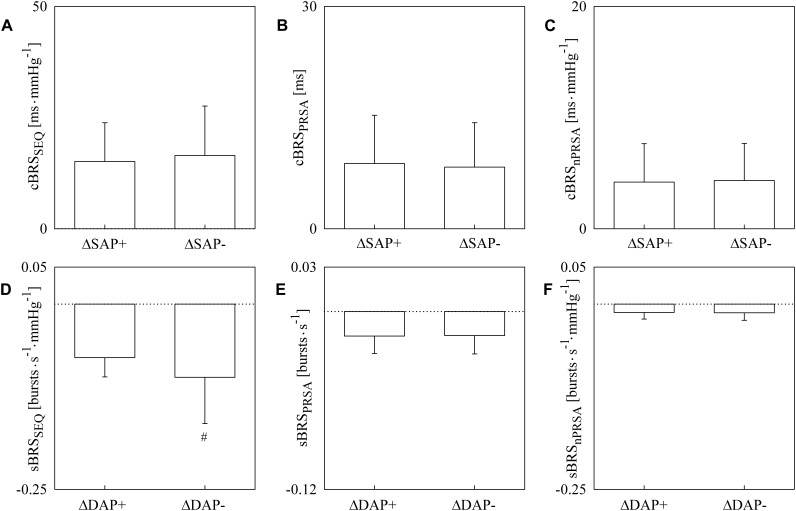
The simple error bar graphs show cBRS **(A–C)** and sBRS **(D–F)** in young healthy subjects undergoing incremental head-up tilt as a function of the sign of, respectively, SAP **(A–C)** and DAP **(D–F)** variations. cBRS and sBRS were estimated using three different approaches, namely SEQ **(A,D)**, PRSA **(B,E)**, and nPRSA **(C,F)** methods. Data were pooled together regardless of the experimental condition (i.e., T0, T20, T30, T40, and T60). The results are presented as mean plus standard deviation. The symbol # indicates *p* < 0.05 versus positive AP variations.

The simple error bar graphs of Figure 1 show cBRS (Figure 1A–C) and sBRS (Figure 1D–F) estimates as a function of the type of AP variation, namely positive or negative SAP and DAP change in Figure 1A–F, respectively. cBRS and sBRS are estimated via SEQ (Figure 1A,D), PRSA (Figure 1B,E), and nPRSA (Figure 1C,F) techniques. Data are pooled together regardless of the experimental condition (i.e., T0, T20, T30, T40, and T60) and reported as mean plus standard deviation. cBRS markers were similar when computed over positive and negative SAP variations regardless of the method utilized to estimate cBRS (Figure 1A–C). Conversely, sBRS was more negative when computed over negative than positive DAP variations (Figure 1D). However, this result was obtained exclusively using the SEQ method, while PRSA and nPRSA approaches were not able to differentiate sBRS according to the sign of the DAP change (Figure 1E,F). Results given in Figure 1. are summarized in Table 1 as well.

**Table 1:** cBRS and sBRS as a function of the method and sign of AP variation.

Index	ΔAP+	ΔAP-
cBRS_SEQ_ [ms⋅mmHg^-1^]	15.11 ± 8.74	16.45 ± 11.12
cBRS_PRSA_ [ms]	8.79 ± 6.51	8.31 ± 5.99
cBRS_nPRSA_ [ms⋅mmHg^-1^]	4.20 ± 3.45	4.336 ± 3.33
sBRS_SEQ_ [bursts⋅s^-1^⋅mmHg^-1^]	-0.072 ± 0.026	-0.099 ± 0.062^#^
sBRS_PRSA_ [bursts⋅s^-1^]	-0.017 ± 0.012	-0.016 ± 0.012
sBRS_nPRSA_ [bursts⋅s^-1^⋅mmHg^-1^]	-0.011 ± 0.009	-0.012 ± 0.010


**AP, arterial pressure; BR, baroreflex; cBR, cardiac BR; sBR, sympathetic BR; cBRS, cBR sensitivity; sBRS, sBR sensitivity; ΔAP+, positive AP variation; ΔAP-, negative AP variation; SEQ, sequence method; PRSA, phase rectified signal averaging method; nPRSA, normalized PRSA. Data are presented as mean ± standard deviation. The symbol # indicates p < 0.05 versus ΔAP+. ΔAP is intended as ΔSAP for the computation of cBRS and ΔDAP for the computation of sBRS*.*

**FIGURE 2:**
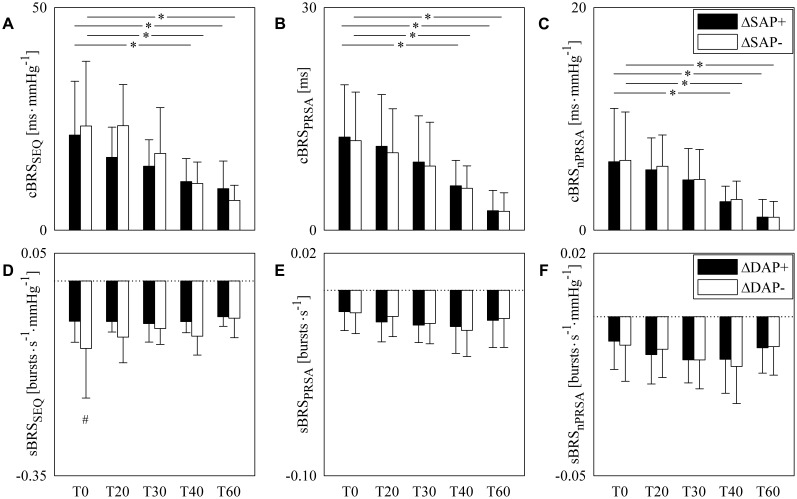
The grouped error bar graphs show cBRS **(A–C)** and sBRS **(D–F)** in young healthy subjects undergoing incremental head-up tilt as a function of the experimental condition (i.e., T0, T20, T30, T40, and T60). cBRS and sBRS were estimated using three different approaches, namely SEQ **(A,D)**, PRSA **(B,E)**, and nPRSA **(C,F)** methods, and reported by separately considering positive (black bars) and negative (white bars) SAP variations in **(A–C)** and DAP changes in **(D–F)**. The results are presented as mean plus standard deviation. The symbol # indicates a significant change of cBRS **(A–C)**, or sBRS **(D–F)**, versus positive AP variations with *p* < 0.05 within the same experimental condition (i.e., T0, T20, T30, T40, or T60). The symbol ^∗^ indicates a significant change with *p* < 0.05 versus T0 within the same type of cBRS **(A–C)**, or sBRS **(D–F)**.

The grouped error bar graphs of Figure 2 show cBRS (Figure 2A–C) and sBRS (Figure 2D–F) estimates as a function of experimental condition (i.e., T0, T20, T30, T40, and T60). cBRS estimates are differentiated according to the sign of SAP variations, while sBRS are separated according to the direction of DAP changes. In all panels black and white bars indicate BRS estimate computed over, respectively, positive and negative AP variations. cBRS and sBRS are estimated via SEQ (Figure 2A,D), PRSA (Figure 2B,E), and nPRSA (Figure 2C,F) techniques. Data are reported as mean plus standard deviation. Regardless of the method, cBRS markers moved toward 0 with the magnitude of the orthostatic challenge. Significant cBRS decreases were observed with tilt table inclination angles higher than, or equal to, 40° (Figure 2A–C). This result held regardless of the sign of SAP changes utilized to assess cBRS. Remarkably, no significant differences were observed within the same experimental condition between cBRS estimates computed over positive and negative SAP variations. sBRS was more stable with the magnitude of the orthostatic challenge (Figure 2D–F). Indeed, no significant changes versus T0 were observed when sBRS was computed via SEQ, PRSA and nPRSA methods (Figure 2D–F). Remarkably, sBRS computed over negative DAP variations was more negative than that derived from positive DAP changes and this difference was significant at T0 (Figure 2D). The finding was detected only by SEQ method: indeed, when sBRS was computed via PRSA and nPRSA techniques, its value did not depend on the direction of DAP changes in any of the considered experimental conditions (Figure 2E,F). Results given in Figure 2 are summarized in Table 2 as well.

**Table 2:** cBRS and sBRS as a function of the method, sign of AP variation and title table inclination.

Index	T0		T20		T30		T40		T60	
					
	ΔAP+	ΔAP-	ΔAP+	ΔAP-	ΔAP+	ΔAP-	ΔAP+	ΔAP-	ΔAP+	ΔAP-
cBRS_SEQ_ [ms⋅mmHg^-1^]	21.35 ± 12.08	23.39 ± 14.55	16.35 ± 6.78	23.46 ± 9.25	14.38 ± 5.91	17.24 ± 10.29	10.91 ± 5.18^∗^	10.47 ± 4.83^∗^	9.32 ± 6.21^∗^	6.69 ± 3.39^#∗^
cBRS_PRSA_ [ms]	12.54 ± 7.05	12.06 ± 6.55	11.30 ± 6.97	10.43 ± 5.93	9.18 ± 6.23	8.64 ± 5.9	5.97 ± 3.43^∗^	5.64 ± 3.02^∗^	2.62 ± 2.73^∗^	2.54 ± 2.5^∗^
cBRS_nPRSA_ [ms⋅mmHg^-1^]	6.15 ± 4.79	6.27 ± 4.34	5.43 ± 2.86	5.74 ± 2.80	4.52 ± 2.81	4.54 ± 2.71	2.56 ± 1.39^∗^	2.75 ± 1.65^∗^	1.17 ± 1.59^∗^	1.16 ± 1.42^∗^
sBRS_SEQ_ [bursts⋅s^-1^⋅mmHg^-1^]	-0.072 ± 0.038	-0.121 ± 0.089^#^	-0.073 ± 0.019	-0.101 ± 0.046	-0.077 ± 0.033	-0.085 ± 0.028	-0.073 ± 0.020	-0.099 ± 0.034	-0.064 ± 0.017	-0.067 ± 0.035
sBRS_PRSA_ [bursts⋅s^-1^]	-0.011 ± 0.010	-0.012 ± 0.011	-0.017 ± 0.011	-0.014 ± 0.011	-0.019 ± 0.009	0.018 ± 0.011	-0.020 ± 0.014	-0.022 ± 0.014	-0.016 ± 0.015	-0.015 ± 0.015
sBRS_nPRSA_ [bursts⋅s^-1^⋅mmHg^-1^]	-0.008 ± 0.009	-0.009 ± 0.011	-0.012 ± 0.009	-0.010 ± 0.009	-0.014 ± 0.007	-0.014 ± 0.009	-0.013 ± 0.011	-0.016 ± 0.012	-0.010 ± 0.008	-0.009 ± 0.009


**AP, arterial pressure; BR, baroreflex; cBR, cardiac BR; sBR, sympathetic BR; cBRS, cBR sensitivity; sBRS, sBR sensitivity; ΔAP+, positive AP variation; ΔAP-, negative AP variation; SEQ, sequence method; PRSA, phase rectified signal averaging method; nPRSA, normalized PRSA. Data are presented as mean ± standard deviation. The symbol ^∗^ indicates p < 0.05 versus T0 within the same type of ΔAP variation. The symbol # indicates p < 0.05 versus ΔAP+ within the same experimental condition. ΔAP is intended as ΔSAP for the computation of cBRS and ΔDAP for the computation of sBRS*.*

**FIGURE 3:**
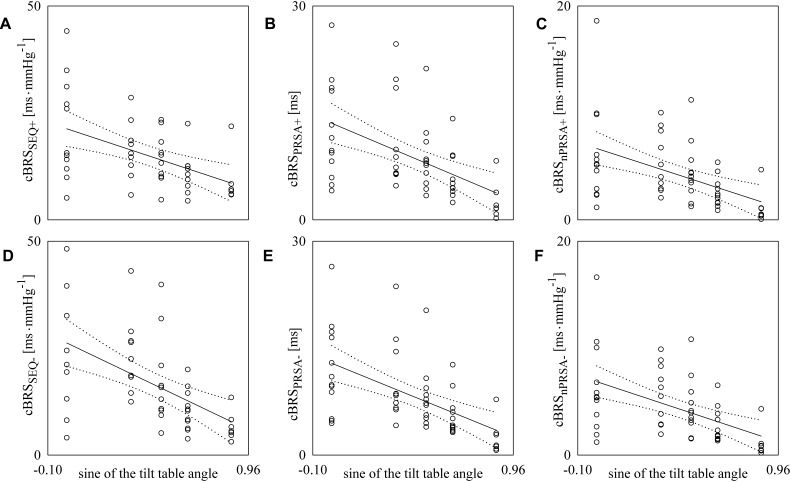
The scatter plots show the results of the linear correlation analysis between cBRS estimates and the sine of the tilt table angles. Each circle represents the cBRS estimate computed in a subject in the assigned experimental condition. cBRS was estimated via SEQ **(A,D)**, PRSA **(B,E)**, and nPRSA **(C,F)** methods. The cBRS estimates were obtained by separately considering positive **(A–C)** and negative **(D–F)** SAP variations. The linear regression line (solid line) and its 95% confidence interval (dotted lines) are plotted only if the Pearson correlation coefficient is significantly different from 0 with *p* < 0.05.

Figure 3 reports the scatter plots of the cBRS on the magnitude of the orthostatic challenge quantified by the sine of the tilt table angles. Each open circle represents the cBRS computed in a specific subject in a given experimental condition. cBRS is estimated according to SEQ (Figure 3A,D), PRSA (Figure 3B,E), and nPRSA (Figure 3C,F) techniques. Panels on the top (Figure 3A–C) are relevant to cBRS computed over positive SAP changes, while those at the bottom (Figure 3D–F) are relevant to cBRS calculated over negative SAP changes. The linear regression (solid line) is drawn along with its 95% confidence interval (dotted lines) if a significant linear association between the two variables was found. All the cBRS estimates were significantly and negatively correlated with the magnitude of the orthostatic challenge regardless of the method and sign of the SAP variation. Pearson correlation coefficient *r* and type I error probability *p* were *r* = -0.486; *p* = 7.16⋅10^-4^ (Figure 2A), *r* = -0.518; *p* = 9.74⋅10^-5^ (Figure 2B), *r* = -0.481; *p* = 3.15⋅10^-4^ (Figure 2C), *r* = -0.531; *p* = 1.22⋅10^-4^ (Figure 2D), *r* = -0.545; *p* = 3.56⋅10^-5^ (Figure 2E), and *r* = -0.509; *p* = 1.36⋅10^-4^ (Figure 2F).

**FIGURE 4:**
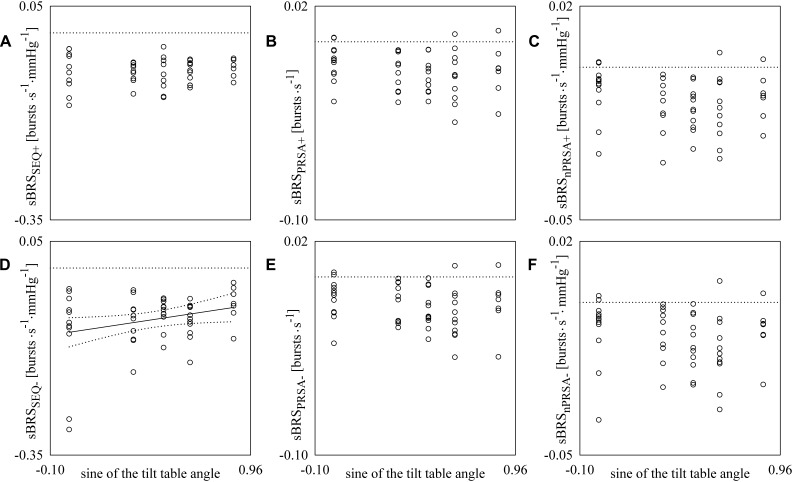
The scatter plots show the results of the linear correlation analysis between sBRS estimates and the sine of the tilt table angles. Each circle represents the sBRS estimate computed in a subject in the assigned experimental condition. sBRS was estimated via SEQ **(A,D)**, PRSA **(B,E)**, and nPRSA **(C,F)** methods. The sBRS estimates were obtained by separately considering positive **(A–C)** and negative **(D–F)** DAP variations. The linear regression line (solid line) and its 95% confidence interval (dotted lines) are plotted only if the Pearson correlation coefficient is significantly different from 0 with *p* < 0.05.

Figure 4 has the same structure as Figure 3 but reports sBRS as a function of the sine of the tilt table angles. A linear association of sBRS derived from SEQ method with the magnitude of the orthostatic challenge was detected only when sBRS was assessed over negative DAP changes (Figure 4D). Pearson correlation coefficient *r* was positive (i.e., *r* = 0.286) and type I error probability *p* was 4.43⋅10^-2^. Conversely, no significant linear association was found when sBRS was derived from the SEQ method over positive DAP variations (Figure 4A). The same conclusion was drawn when sBRS was estimated via PRSA and nPRSA techniques and held regardless of the sign of the DAP changes (Figure 4B,C,E,F).

**FIGURE 5:**
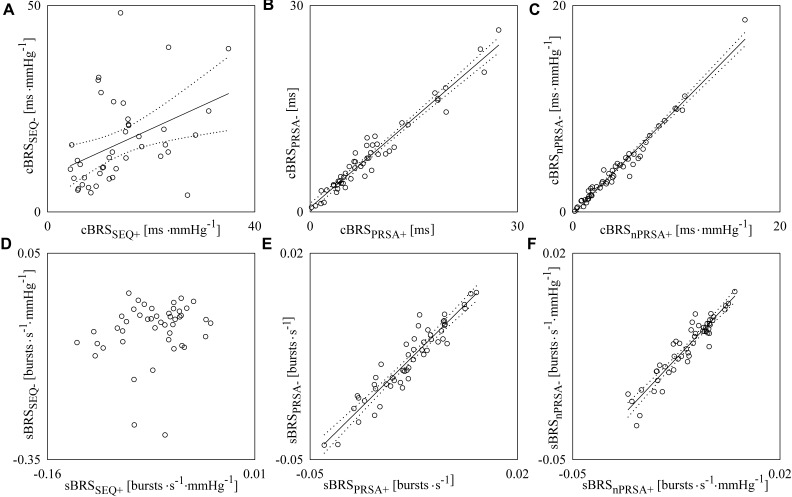
The scatter plots show the results of linear correlation analysis in the planes (cBRS_SEQ+_, cBRS_SEQ-_) **(A)**, (cBRS_PRSA+_, cBRS_PRSA-_) **(B)** and (cBRS_nPRSA+_, cBRS_nPRSA-_) **(C)**, (sBRS_SEQ+_, sBRS_SEQ-_) **(D)**, (sBRS_PRSA+_, sBRS_PRSA-_) **(E)**, and (sBRS_nPRSA+_, sBRS_nPRSA-_) **(F)** in young healthy subjects undergoing incremental head-up tilt. Each circle represents the pair of cBRS **(A–C)** or sBRS **(D–F)** estimates computed in a subject in a given experimental condition. Data were pooled together regardless of the experimental condition (i.e., T0, T20, T30, T40, and T60). The linear regression line (solid line) and its 95% confidence interval (dotted lines) are plotted only if the Pearson correlation coefficient is significantly different from 0 with *p* < 0.05.

Figure 5 reports the scatter plots of the cBRS derived from negative SAP variations on that obtained from positive SAP changes (Figure 5A–C) and the scatter plots of the sBRS derived from negative DAP variations on that obtained from positive DAP changes (Figure 5D–F). Each open circle represents a pair of cBRS (Figure 5A–C), or sBRS (Figure 5D–F), estimates computed in a specific subject in a given experimental condition. cBRS and sBRS are estimated according to SEQ (Figure 5A,D), PRSA (Figure 5B,E), and nPRSA (Figure 5C,F) methods. Data are pooled together regardless of the experimental condition. The linear regression (solid line) is drawn along with its 95% confidence interval (dotted lines) if a significant linear association between the two variables was found. A significant positive association between cBRS computed over positive and negative SAP variations was found regardless of the method (Figure 5A–C): the Pearson correlation coefficient *r* and the type I error probability *p* were 0.413 and 6.62⋅10^-3^ in Figure 5A, 0.974 and 1.87⋅10^-33^ in Figure 5B, and 0.985 and 6.04⋅10^-39^ in Figure 5C. Conversely, a significant linear association between sBRS calculated over positive and negative DAP changes was detected only when sBRS was derived via PRSA and nPRSA techniques (i.e., *r* = 0.943, *p* = 5.65⋅10^-25^ in Figure 5E and *r* = 0.938, *p* = 3.11⋅10^-24^ in Figure 5F). No significant correlation was found between sBRS computed over positive and negative DAP variations via the SEQ method (Figure 5D).

Results remained valid when gender disproportion was fixed by considering only females (see Supplementary Tables S1, S2). Remarkably, the exclusion of the three men reduced the age dispersion to 20–28 years (min–max range, median = 22 years), thus limiting the impact of age spreading. Moreover, findings of the SEQ method were confirmed (see Supplementary Tables S3, S4) when prerequisites for selection of joint SEQ+ and SEQ- patterns were applied according to standard setting of minimal absolute total variations of SAP or DAP, minimal absolute total variations of HP or MSNA burst rate and minimal absolute value of the correlation coefficient.

## Discussion

The main findings of this work can be summarized as follows: (i) the asymmetry of the cBR is not detectable from spontaneous fluctuations of HP and SAP during incremental head-up tilt maneuver; (ii) the asymmetry of the sBR is detectable from spontaneous variations of MSNA burst rate and DAP at rest in supine conditions and it is lost in response to the sympathetic activation and vagal withdrawal induced by the postural challenge; (iii) the SEQ method is much more powerful than the PRSA technique in describing the cBR and sBR hysteresis.

### The cBR Hysteresis Is Not Detectable From Spontaneous Variability of SAP and HP

The cBR responds to an AP drop with an HP shortening and to an AP rise with an HP lengthening (Smyth et al., 1969). The cBR is traditionally characterized through an interventional approach imposing a large AP drop or rise via the administration of a vasoactive drug (Smyth et al., 1969; Pickering et al., 1972) or the stimulation of the barosensory areas in the carotid arteries via a neck chamber (Eckberg, 1980). In the interventional analysis the gain of the HP-AP relation, usually referred to as cBRS, was obtained as the slope of the linear regression of HP on SAP. Linear relation is estimated starting from the highest SAP value just after the intervention and ending to the SAP nadir in the case of induced SAP falls or starting from the lowest SAP value just after the intervention and ending to the SAP peak in the case of induced SAP rises. cBRS is non-negative because cBR buffers SAP changes with parallel HP variations and a migration of cBRS toward 0 indicates a weak buffering. The cBR exhibits an asymmetric behavior resulting from the dependence of the cBRS on the sign of the SAP changes: the linear portion of the relation of HP on SAP is steeper when SAP is rising than falling. This feature leads to a longer HP just after a SAP change immediately followed by an opposite sign SAP variation of the same absolute magnitude (Pickering et al., 1972; Rudas et al., 1999; Studinger et al., 2007). As a consequence, the trajectory followed by the point in the plane (SAP, HP) is not a straight line, even for small variations of SAP, but an elliptical HP-SAP pattern. This typical phenomenon is termed hysteresis (Studinger et al., 2007; Hart et al., 2011). The cBR hysteresis suggests that cBR buffers more efficiently SAP increases than decreases. The asymmetry of the cBR originates from the viscoelastic properties of the barosensory vessels, as assessed from the diameter-pressure relation, leading to larger carotid artery diameter changes, and consequently to a greater stretch of the barosensitive vessels, when SAP is rising than falling (Bonyhay et al., 1997; O’Leary et al., 2005). However, it was suggested that the asymmetric behavior of the diameter-pressure relation could not be the sole mechanism responsible for the cBR hysteresis. Indeed, an important role is played by the asymmetric behavior of the neural component as assessed from the HP-diameter relation (Studinger et al., 2007). While cBR hysteresis was frequently studied using interventional analysis (Pickering et al., 1972; Rudas et al., 1999; Studinger et al., 2007), the cBRS was rarely computed by separately considering positive and negative SAP variations over spontaneous fluctuations of HP and SAP (Parati et al., 1988; Martin-Vazquez and Reyes Del Paso, 2010; Davydov et al., 2018; De Maria et al., 2018). This lack is much more evident when searching for studies featuring the contemporaneous application of different BR characterization methods and an analysis over multiple BR arms. In the present study, SEQ and PRSA techniques were exploited for the analysis of the cBR hysteresis from spontaneous fluctuations of SAP and HP. The SEQ method, scanning HP and SAP variabilities to search for short sequences of assigned length featuring consecutive and parallel variations of SAP and HP, was applied by separately considering positive and negative changes (Bertinieri et al., 1985; De Maria et al., 2018). The PRSA approach, usually anchoring the analysis of HP variability to a specific direction of the SAP changes (i.e., positive) (Bauer et al., 2010; Muller et al., 2012), was applied even in the opposite direction (i.e., negative) (De Maria et al., 2018). We found no significant dependency of the cBRS over the direction of SAP changes when both the data were pooled together regardless of the experimental condition and they were analyzed separately in each experimental session. The inability of cBRS estimates based on spontaneous fluctuations to detect the asymmetric behavior of the cBR might be related to the smallness of the SAP changes that are insufficient for exploring portions of the HP-SAP relation with significantly different slopes. The lack of the cBR asymmetry was confirmed even when the slopes of the linear regression of cBRS estimated over positive and negative SAP variations on the sine of the tilt table angle were compared: indeed, regardless of the method utilized to extract cBRS, the slopes were similar, thus suggesting that both cBRS estimates computed over positive and negative SAP changes contribute equally in closed loop conditions to the decrease of cBRS with the magnitude of the orthostatic challenge (Cooke et al., 1999; Furlan et al., 2000; O’Leary et al., 2003; Dalla Vecchia et al., 2013; Marchi et al., 2016b; De Maria et al., 2018). We advocate the assessment of the impact of HP-SAP causality (Porta et al., 2000) on this conclusion: a possibility is to limit the eventual effect of HP variability rhythms that might be of origin different from cBR, such as the respiratory sinus arrhythmia, via low-pass filtering the series (Oosting et al., 1997).

### The sBR Hysteresis Is Detectable From Spontaneous Variability of DAP and MSNA Burst Rate

The sBR buffers DAP changes by reducing the probability of observing a MSNA burst when AP is high and by increasing it when DAP is low. The probability is usually expressed as the number of MSNA bursts per 100 cardiac beats, termed burst incidence (Sundlof and Wallin, 1978; Ebert and Cowley, 1992; Ichinose et al., 2004; Keller et al., 2006; Taylor et al., 2015) or percentage, termed burst threshold (Kienbaum et al., 2001; Hart et al., 2010, 2011; Barbic et al., 2015). This MSNA-DAP relation holds as well when total MSNA (Matsukawa et al., 1996; O’Leary et al., 2003), or total MSNA per cardiac beat (Halliwill et al., 1996; Ichinose et al., 2004; Fu et al., 2006; Dutoit et al., 2010), or total MSNA per 100 beats (Studinger et al., 2009), or parameters describing the MSNA burst strength, such as the average amplitude (Sundlof and Wallin, 1978; Ebert and Cowley, 1992; Taylor et al., 2015) or area of the MSNA burst (Rudas et al., 1999; Kienbaum et al., 2001; Ichinose et al., 2004; O’Leary et al., 2005; Keller et al., 2006), were considered. The sBR was first typified by exploiting the spontaneous fluctuations of DAP and MSNA in absence of any pharmacological intervention inducing AP rises or falls (Sundlof and Wallin, 1978) and later by applying pharmacological approaches (Ebert and Cowley, 1992; Halliwill et al., 1996; Matsukawa et al., 1996; Rudas et al., 1999; Studinger et al., 2009; Dutoit et al., 2010; Hart et al., 2010). In the sBR analysis, regardless of the exploitation of spontaneous or pharmacological approach, the gain of the MSNA-AP relation, usually referred to as sBRS, was obtained as the slope of the linear regression of burst incidence, burst strength, or total MSNA at a given DAP level on DAP value. sBRS is non-positive because it is less likely to find a MSNA burst associated with higher DAP values and a migration of sBRS toward 0 indicates a reduced buffering of DAP variations with appropriate MSNA modifications. Like the cBR, the sBR exhibits an asymmetric behavior resulting from the dependence of the sBRS on the sign of the DAP changes: indeed, the linear portion of the relation of probability of finding a MSNA burst at an assigned DAP level on DAP is steeper when DAP is falling than rising. This feature leads to a lower likelihood of MSNA burst just after a DAP change immediately followed by an opposite sign DAP variation of the same absolute magnitude (Sundlof and Wallin, 1978; Studinger et al., 2009; Hart et al., 2011). This phenomenon is referred to as sBR hysteresis in analogy to the cBR one (Studinger et al., 2009; Hart et al., 2011) and suggests that sBR buffers more efficiently DAP decreases than increases. Remarkably, the sBR hysteresis was found to be evident when both spontaneous (Sundlof and Wallin, 1978; Hart et al., 2011) and pharmacologically driven (Studinger et al., 2009) MSNA and DAP variations were considered. Moreover, it was more easily detectable via burst incidence parameters (Sundlof and Wallin, 1978; Studinger et al., 2009; Hart et al., 2011) than via burst strength markers (Rudas et al., 1999; O’Leary et al., 2005). In this study, we applied a recently proposed dynamical approach to the characterization of sBR (Marchi et al., 2016b) that appears also suitable for the assessment of sBR hysteresis. This analysis exploits the definition of MSNA variability representing the variations of MSNA burst rate over time (Marchi et al., 2016a), scans MSNA and DAP series to search for short antiparallel joint ramps, and computes sBRS as the average slope of the regression line of MSNA burst rate on DAP over these joint MSNA-DAP patterns. In the present study, this approach was applied by separately considering joint MSNA-DAP patterns featuring positive and negative DAP variations. Also, the PRSA approach (Bauer et al., 2010; Muller et al., 2012) was applied to the MSNA burst rate variability series and its analysis was anchored separately to positive and negative DAP changes (De Maria et al., 2018). Given that SEQ and PRSA methods do not require pharmacological challenges they provide an alternative to pharmacological methods for the assessment of the sBR hysteresis (Rudas et al., 1999; Studinger et al., 2009). SEQ and PRSA techniques provide an alternative to non-pharmacological methods for the evaluation of sBR asymmetry as well (Hart et al., 2011). Indeed, the traditional method based on spontaneous variability (Kienbaum et al., 2001) is useless to study the sBR hysteresis because it does not account for MSNA-DAP causal interactions along sBR (i.e., the association between the MSNA burst rate and DAP could occur according to schemes not necessarily linking MSNA burst rate increases to DAP decreases and *vice versa*). As a consequence, the traditional method (Kienbaum et al., 2001) was adapted in Hart et al. (2011) to embed the flow of time in the analysis by conditioning the probability of occurrence of the MSNA burst at a given DAP to the sign of the DAP modification. However, this strategy reduces the reliability of the sBRS estimate, especially over short data sequences, as a consequence of the reduced consistency of the estimate of the probability of observing an MSNA burst at given DAP when the direction of the DAP change is accounted for.

In our study, when data were pooled together regardless of the experimental condition, we found a significant dependency of the sBRS on the direction of DAP changes with more negative sBRS values while DAP falling than rising. Therefore, our dynamical approach to the characterization of sBR from spontaneous variability confirms the findings obtained via static non-pharmacological and pharmacological approaches (Sundlof and Wallin, 1978; Studinger et al., 2009; Hart et al., 2011). When the experimental conditions were considered separately, this result was significant only in supine condition. Indeed, during the orthostatic challenge and the consequent increase of MSNA burst rate (Cooke et al., 1999; Furlan et al., 2000; O’Leary et al., 2003; Marchi et al., 2016a), the asymmetric behavior of the sBR was no longer evident. This finding is not surprising given that the sBR asymmetry depends on the MSNA burst rate and was lost when the mean MSNA burst rate is increased (i.e., sBR tends to improve its ability to respond to DAP rises than falls at high MSNA levels) (Sundlof and Wallin, 1978; Rudas et al., 1999; Studinger et al., 2009; Hart et al., 2011). The different behavior of sBR in response to DAP ups and downs was stressed by the separate analysis of the dependency of sBRS on sine of the tilt table angle. Indeed, the positive linear relation of sBRS on the magnitude of the orthostatic challenge, reported in Marchi et al. (2016b) when sBRS was assessed regardless of the direction of the DAP changes, was confirmed in our study only when sBRS was evaluated over DAP drops. It seems that the ability to counteract DAP falls was more and more reduced with tilt table angles, while that to counteract DAP rises was preserved. This finding might result in a reduced tolerance of compensating AP drops during the postural challenge at high tilt table inclination. However, even though the tendency to move toward 0 with the magnitude of the challenge is confirmed (Marchi et al., 2016b), we note that this tendency did not produce a significant variation of the sBRS. This result supports the notion of a preserved sBRS with the magnitude of the hypovolemic challenge (Ichinose et al., 2004; Barbic et al., 2015) and suggests a general maintenance of sBR control (Porta et al., 2017). This result is in disagreement with studies assessing sBRS during head-up tilt (Halliwill et al., 1996; Fu et al., 2006). Indeed, these studies observed more negative sBRS values during the postural challenge compared to baseline but sBRS was expressed in different units of measurement (i.e., arbitrary units per mmHg), thus suggesting a possible dependency of the conclusions on the type of the monitored quantity (Kienbaum et al., 2001; Keller et al., 2006) and stressing the importance of using approaches that are independent of normalizing factors (Marchi et al., 2016b) necessary to compensate MSNA amplitude parameters for the variable proximity of the electrode to the nerve fascicle (Sundlof and Wallin, 1978; Kienbaum et al., 2001). A similar observation holds when comparing studies supporting (Sundlof and Wallin, 1978; Hart et al., 2011) and not supporting (Rudas et al., 1999; O’Leary et al., 2005) the existence of sBR hysteresis.

### The SEQ Method Is More Powerful Than the PRSA Technique in Assessing Hysteresis of BR Arms

Both the SEQ and PRSA methods (Bertinieri et al., 1985; Bauer et al., 2010; Muller et al., 2012) can provide BRS estimates by differentiating positive and negative AP variations (De Maria et al., 2018). However, we confirm that SEQ and PRSA methods cannot be considered equivalent (De Maria et al., 2018). Indeed, the correlations between cBRS estimates computed over positive and negative SAP variations via the PRSA and nPRSA methods are much stronger than those observed through the SEQ technique, as suggested by the wider scattering in Figure 5A than in Figure 5B,C. This behavior has been interpreted as a hallmark of a greater rigidity of the PRSA methods compared to the SEQ one in evaluating the gain of the HP-SAP relation from spontaneous fluctuations of HP and SAP variables (De Maria et al., 2018). This conclusion is strengthened in the present study by the analysis of the sBR. Indeed, while the sBRS estimates derived from positive and negative DAP variations computed via the SEQ method are uncorrelated, those derived from PRSA techniques are again strictly correlated. However, the uncorrelation between the sBRS estimates detected by the SEQ analysis is expected, given that the sBR hysteresis detected from the spontaneous fluctuations of DAP and MSNA burst rate is likely to limit the correlation between sBRS markers. Therefore, the strong correlation between sBRS derived from positive and negative DAP variations pointed out by PRSA techniques seems to be artificial. The higher rigidity of the PRSA techniques might have contributed to the inability of these methods to suggest the asymmetric behavior of the sBR. Therefore, we recommend the use of the SEQ method for the assessment of the BR hysteresis from spontaneous fluctuations of cardiovascular variables because the SEQ method is more prone to provide independent descriptors of the BR functioning when this reflex is solicited by positive and negative AP variations. The better performance of the SEQ method may lie in its focus on particularly rare patterns lasting several beats and more likely of BR origin, while the PRSA utilizes basic AP variations from one beat to the next one that might not necessarily drive HP or MSNA responses. Conversely, the PRSA method should be preferred for its robustness and repeatability of the results, when BRS estimates are assessed regardless of the sign of the AP variations (Bauer et al., 2010; Maestri et al., 2017; Pinna et al., 2017).

## Conclusion

Two dynamical approaches for the characterization of the BR hysteresis, namely the SEQ and PRSA methods, from spontaneous fluctuations of cardiovascular variables were applied simultaneously to cBR and sBR. We recommend the use of the SEQ method for future studies on the BR hysteresis given that this approach is much more powerful than the PRSA technique in typifying the different responses of BR arms to positive and negative AP spontaneous changes. The expected asymmetric behavior of sBR and cBR was detected exclusively in the sBR and in absence of an orthostatic stimulus, thus stressing the much more inherent asymmetry, and non-linearity, of the sBR visible even for small variations of AP and in unstressed conditions. The detection of sBR asymmetry demonstrates that the BR hysteresis phenomenon can be studied from spontaneous fluctuations of cardiovascular variables, thus prompting for the application of this approach to other branches of BR, such as the peripheral resistance (Porta et al., 2018a) and stroke volume (Casadei et al., 1992). Moreover, the different behavior between cBR and sBR makes evident the complementary information that can be derived from the simultaneous characterization of different arms of the BR control. We advocate future applications in healthy subjects under different experimental challenges and in a more numerous healthy group to confirm the inability of detecting the asymmetric behavior of cBR from spontaneous variability and check whether the eventual maintenance of sBR asymmetry during an orthostatic challenge could identify some pathological conditions, such as orthostatic intolerance. Even though results of this study are confirmed after reducing gender and age variability, the present findings need to be corroborated by specific studies accounting explicitly for age and gender factors (Ng et al., 1993; Laitinen et al., 1998; Fisher et al., 2012). This exploration is important because the majority of the studies present in literature about the modifications of BRS with age and gender does not taken into account the BRS hysteresis phenomenon. At this regard the present approach, fully grounded on spontaneous fluctuations of physiological variables, could be of help by setting an analysis framework useful in retrospective studies.

## Data Availability

The datasets generated for this study are available on request to the corresponding author.

## Author Contributions

BDM analyzed the data. BDM and AP drafted the manuscript. ME and EL performed the experiments. BDM, VB, BC, EV, ME, EL, MB, SC, LDV, and AP interpreted the data, revised the manuscript, and approved the final version of the manuscript.

## Conflict of Interest Statement

The authors declare that the research was conducted in the absence of any commercial or financial relationships that could be construed as a potential conflict of interest.
