# Supplementary material for: Characterization of the Asymmetry of the Cardiac and Sympathetic Arms of the Baroreflex From Spontaneous Variability During Incremental Head-Up Tilt
Source: Front Physiol. 2019 Apr 2;10:342. doi: 10.3389/fphys.2019.00342 (PMC6454064; doi:10.3389/fphys.2019.00342)
Supplement: Supplementary file 1 [file Table_1.DOC]

**Supplementary Material of “Characterization of the asymmetry of the cardiac and sympathetic arms of the baroreflex from spontaneous variability during incremental head-up tilt” by B. De Maria *et al.***

**Effect of gender disproportions and age dispersion**

We examined the impact of the different gender proportions on the conclusions by considering only females (i.e. the 3 men were excluded)). Results are reported in Tabs.S1,S2. This exclusion limited the dispersion of age to 20-28 yrs (min-max, median: 22 yrs) as well.

Table S1 shows cBRS and sBRS estimates as a function of the type of AP variation, namely positive or negative SAP and DAP change respectively. Data are pooled together regardless of the experimental condition. cBRS markers were similar when computed over positive and negative SAP variations regardless of the analytical method. Conversely, sBRS computed by SEQ method was more negative when computed over negative DAP changes than positive DAP variations. No differences were observed when PRSA and nPRSA approaches were considered.

Table S2 shows the results of cBRS and sBRS estimates as a function of the experimental conditions and sign of SAP and DAP variations, respectively. Regardless of the method and sign of SAP variations cBRS markers moved toward 0 with the magnitude of the orthostatic challenge and significant cBRS decreases were observed with tilt table inclination angles of 40° and 60°. No significant differences were observed within the same experimental condition between cBRS estimates computed over positive and negative SAP variations. sBRS did not vary with the magnitude of the orthostatic challenge: no significant changes versus T0 were observed for any of the considered method and sign of DAP variations. Remarkably, when sBRS was assessed by the SEQ method, sBRS computed over negative DAP variations was more negative than that derived from positive DAP changes during T0. Within the experimental condition no asymmetric behavior of sBRS was observed by PRSA and nPRSA methods.

All the cBRS estimates were negatively correlated with the sine of the tilt table angle, independently of the sign of SAP variations and analytical method. The Pearson correlation coefficient *r* and type I error probability *p* were *r*=-0.38; *p*=2.68·10^-2^ for cBRS_SEQ+_, *r*=-0.558; *p*=4.2·10^-4^ for cBRS_SEQ-_, *r*=-0.507; *p*=1.15·10^-3^ for cBRS_PRSA+_, *r*=-0.541; *p*=4.54·10^-4^ for cBRS_PRSA-_, *r*=-0.507; *p*=1.16·10^-3^ for cBRS_nPRSA+_ and *r*=-0.543; *p*=4.28·10^-4^ for cBRS_nPRSA-_.

Only sBRS_SEQ-_ was significantly correlated to the magnitude of the orthostatic challenge and correlation coefficient was positive (*r*=0.334; *p*=4.36·10^-2^). No other significant correlations have been observed.

After pooling together all the data regardless of the experimental condition, a significant positive association between cBRS computed over positive and negative SAP variations was found. This conclusion held regardless of the method. Pearson correlation coefficient *r* and type I error probability *p* were 0.473 and 6.25·10^-3^ for SEQ method, 0.982 and 8.29·10^-28^ for PRSA method and 0.989 and 9.13·10^-32^ for nPRSA method. Conversely, a significant linear association between sBRS calculated over positive and negative DAP changes was detected only when sBRS was derived via PRSA and nPRSA techniques (i.e. *r*=0.938, *p*=3.44·10^-18^ and *r*=0.939, *p*=2.94·10^-18^, respectively). No significant correlation was found between sBRS computed over positive and negative DAP variations via the SEQ method.

These results support that the conclusion drawn in presence of the disproportions of gender and larger interval of age (Tabs.1,2) holds when only females are considered and age dispersion is reduced (Tabs.S1,S2).

**Effect of the application of standard SEQ method thresholds**

We repeated all the SEQ analyses by applying more traditional criteria reported in literature for the definition of patterns of BR origin. As to the cBR characterization we applied the following thresholds (Bertinieri *et al.*, 1985; Parati *et al.*, 1988): i) absolute total HP variation > 5 ms; ii) absolute total SAP variation > 1 mmHg; iii) correlation coefficient computed over the joint HP-SAP pattern > 0.85. As to the sBR characterization we applied the following thresholds (Marchi *et al.*, 2016): i) absolute total MSNA burst rate variation > 0 burst·s^-1^; ii) absolute total DAP variation > 1 mmHg; iii) absolute correlation coefficient computed over joint MSNA-DAP pattern > 0.85. The verification of standard prerequisites was carried out only in the case of the SEQ method because the PRSA approach was already applied as originally proposed in (Bauer et *al.*, 2010; Muller *et al.*, 2012).

Table S3 shows cBRS and sBRS estimates obtained by the SEQ method after checking the prerequisites for the definition of cBR and sBR sequences as a function of the sign of AP variation. Data are pooled together regardless of the experimental condition. cBRS_SEQ_ markers were similar when computed over positive and negative SAP variations, while sBRS_SEQ_ was more negative when computed over negative DAP changes than positive DAP variations.

Table S4 shows the results of cBRS and sBRS estimates obtained by SEQ method after checking the prerequisites for the definition of cBR and sBR sequences as a function of experimental conditions and sign of SAP and DAP variations. cBRS_SEQ_ markers moved toward 0 with the magnitude of the orthostatic challenge and significant cBRS decreases were observed with tilt table inclination angles of 40° and 60° for negative SAP variations and with angle of 60° for positive SAP variations. No significant differences were observed within the same experimental condition between cBRS estimates computed over positive and negative SAP variations. sBRS did not vary with the magnitude of the orthostatic challenge: no significant changes versus T0 were observed. Remarkably, during T0 sBRS computed over negative DAP variations was more negative than that derived from positive DAP changes.

Regardless of the sign of SAP variations, cBRS estimates computed by the SEQ analysis after the application of the prerequisites were negatively correlated with the sine of the tilt table angle. Pearson correlation coefficient *r* and type I error probability *p* were *r*=-0.468 and *p*=9.2·10^-4^ for cBRS_SEQ+_ and *r*=-0.526 and *p*=1.48·10^-4^ for cBRS_SEQ-_.

sBRS estimates computed by the SEQ analysis after checking the prerequisites were significantly correlated to the sine of tilt table angle when they were calculated over joint SEQ- patterns. The correlation coefficient was positive (*r*=0.374; *p*=7.45·10^-3^). Conversely, sBRS_SEQ+_ assessed after the application of standard thresholds was uncorrelated with tilt table angles.

After pooling together all the data obtained after the application of standard prerequisites for SEQ analysis regardless of the experimental condition, a significant positive association between cBRS_SEQ_ computed over positive and negative SAP variations was found (*r*=0.393; *p*=8.38·10^-3^). Conversely, no significant correlation was found between sBRS_SEQ_ estimates computed over positive and negative DAP variations.

Taking all the results together we can confirm that checking the prerequisites does not modify conclusions likely because the impact of noise of this experimental setup is limited (Porta *et al.*, 2013).

**References**

Bauer, A., Morley-Davies, A., Barthel, P., Muller, A., Ulm, K., Malik, M., et al. (2010). Bivariate phase-rectified signal averaging for assessment of spontaneous baroreflex sensitivity: pilot study of the technology. *J. Electrocardiol.* **43**, 649-653.

Bertinieri, G., di Rienzo, M., Cavallazzi, A., Ferrari, A.U., Pedotti, A., and Mancia, G. (1985). A new approach to analysis of the arterial baroreflex. *J. Hypertens.* **33**, S79-S81.

Marchi, A., Bari, V., De Maria, B., Esler, M., Lambert, E., Baumert, M., et al. (2016). Simultaneous characterization of sympathetic and cardiac arms of the baroreflex through sequence techniques during incremental head-up tilt. *Front. Physiol.* **7**, 438.

Muller, A., Morley-Davies, A., Barthel, P., Hnatkova, K., Bauer, A., Ulm, K., et al. (2012). Bivariate phase-rectified signal averaging for assessment of spontaneous baroreflex sensitivity: normalization of the results. *J. Electrocardiol.* **45**, 77-81.

Parati, G., Di Rienzo, M., Bertinieri, G., Pomidossi, G., Casadei, R., Groppelli, A., et al. (1988). Evaluation of the baroreceptor-heart rate reflex by 24-hour intra-arterial blood pressure monitoring in humans. *Hypertension* **12**, 214-222.

Porta, A., Bari, V., Bassani, T., Marchi, A., Pistuddi, V., and Ranucci, M. (2013). Model-based causal closed-loop approach to the estimate of baroreflex sensitivity during propofol anesthesia in patients undergoing coronary artery bypass graft. *J. Appl. Physiol.* **115**, 1032-1042.

**TABLE S1. cBRS and sBRS only considering females (n=9) as a function of the method and** sign of AP variation.

| Index | ΔAP+ | ΔAP- |
| --- | --- | --- |
| cBRS_SEQ_ [ms·mmHg^-1^] | 15.04±8.93 | 17.79±11.85 |
| cBRS_PRSA_ [ms] | 9.15±6.68 | 8.47±6.04 |
| cBRS_nPRSA_ [ms·mmHg^-1^] | 4.58±3.66 | 4.70±3.52 |
| sBRS_SEQ_ [bursts·s^-1^·mmHg^-1^] | -0.076±0.027 | -0.106±0.071# |
| sBRS_PRSA_ [bursts·s^-1^] | -0.014±0.011 | -0.014±0.011 |
| sBRS_nPRSA_ [bursts·s^-1^·mmHg^-1^] | -0.010±0.009 | -0.011±0.011 |

AP: arterial pressure; BR: baroreflex; cBR: cardiac BR; sBR: sympathetic BR; cBRS: cBR sensitivity; sBRS: sBR sensitivity; ΔAP+: positive AP variation; ΔAP-: negative AP variation; SEQ: sequence method; PRSA: phase rectified signal averaging method; nPRSA: normalized PRSA. Data are presented as mean±standard deviation. The symbol # indicates p<0.05 versus ΔAP+. ΔAP is intended as ΔSAP for the computation of cBRS and ΔDAP for the computation of sBRS.

**TABLE S2. cBRS and sBRS only considering females (n=9) as a function of the method, s**ign of AP variation and title table inclination.

| Index | T0 | | T20 | | T30 | | T40 | | T60 | |
| --- | --- | --- | --- | --- | --- | --- | --- | --- | --- | --- |
|  | ΔAP+ | ΔAP- | ΔAP+ | ΔAP- | ΔAP+ | ΔAP- | ΔAP+ | ΔAP- | ΔAP+ | ΔAP- |
| cBRS_SEQ_  [ms·mmHg^-1^] | 20.21±13.21 | 25.81±13.49 | 14.38±6.18 | 24.66±11.11 | 14.97±6.52 | 18.71±10.91 | 11.69±5.28* | 10.10±5.28* | 10.42±7.64* | 7.39±3.87* |
| cBRS_PRSA_  [ms] | 13.54±7.77 | 12.89±7.16 | 11.00±5.68 | 9.71±3.98 | 9.43±6.93 | 8.79±6.44 | 5.94±3.85* | 5.61±3.11* | 3.30±2.99* | 2.81±2.89* |
| cBRS_nPRSA_  [ms·mmHg^-1^] | 7.03±5.24 | 7.17±4.66 | 5.54±2.49 | 5.73±2.54 | 4.77±3.07 | 4.86±2.92 | 2.70±1.54* | 2.93±1.80* | 1.53±1.79* | 1.37±1.65* |
| sBRS_SEQ_  [bursts·s^-1^·mmHg^-1^] | -0.081±0.037 | -0.145±0.130# | -0.077±0.02 | -0.115±0.048 | -0.083±0.036 | -0.089±0.030 | -0.071±0.020 | -0.104±0.039 | -0.068±0.019 | -0.067±0.042 |
| sBRS_PRSA_  [bursts·s^-1^] | -0.011±0.011 | -0.012±0.012 | -0.014±0.010 | -0.012±0.009 | -0.019±0.010 | -0.017±0.012 | -0.015±0.013 | -0.018±0.013 | -0.012±0.012 | -0.010±0.010 |
| sBRS_nPRSA_  [bursts·s^-1^·mmHg^-1^] | -0.008±0.010 | -0.009±0.013 | -0.011±0.010 | -0.010±0.009 | -0.014±0.008 | -0.013±0.010 | -0.011±0.010 | -0.013±0.012 | -0.007±0.007 | -0.006±0.006 |

AP: arterial pressure; BR: baroreflex; cBR: cardiac BR; sBR: sympathetic BR; cBRS: cBR sensitivity; sBRS: sBR sensitivity; ΔAP+: positive AP variation; ΔAP-: negative AP variation; SEQ: sequence method; PRSA: phase rectified signal averaging method; nPRSA: normalized PRSA. Data are presented as mean±standard deviation. The symbol * indicates p<0.05 versus T0 within the same type of ΔAP variation. The symbol # indicates p<0.05 versus ΔAP+ within the same experimental condition. ΔAP is intended as ΔSAP for the computation of cBRS and ΔDAP for the computation of sBRS.

**TABLE S3. cBRS and sBRS computed via SEQ method after the application of the prerequisites** as a function of the sign of AP variation.

| Index | ΔAP+ | ΔAP- |
| --- | --- | --- |
| cBRS_SEQ_ [ms·mmHg^-1^] | 15.33±8.66 | 16.45±11.01 |
| sBRS_SEQ_ [bursts·s^-1^·mmHg^-1^] | -0.076±0.028 | -0.01±0.065# |

AP: arterial pressure; BR: baroreflex; cBR: cardiac BR; sBR: sympathetic BR; cBRS: cBR sensitivity; sBRS: sBR sensitivity; ΔAP+: positive AP variation; ΔAP-: negative AP variation; SEQ: sequence method. Data are presented as mean±standard deviation. The symbol # indicates p<0.05 versus ΔAP+. ΔAP is intended as ΔSAP for the computation of cBRS and ΔDAP for the computation of sBRS.

**TABLE S4. cBRS and sBRS computed via SEQ method after the application of the prerequisites as a function of t**he method, sign of AP variation and title table inclination.

| Index | T0 | | T20 | | T30 | | T40 | | T60 | |
| --- | --- | --- | --- | --- | --- | --- | --- | --- | --- | --- |
|  | ΔAP+ | ΔAP- | ΔAP+ | ΔAP- | ΔAP+ | ΔAP- | ΔAP+ | ΔAP- | ΔAP+ | ΔAP- |
| cBRS_SEQ_  [ms·mmHg^-1^] | 21.12±11.74 | 23.22±14.30 | 15.73±6.80 | 22.32±9.72 | 15.84±6.37 | 17.80±10.15 | 13.32±6.82 | 10.68±4.99* | 7.86±4.40* | 6.53±3.64* |
| sBRS_SEQ_  [bursts·s^-1^·mmHg^-1^] | -0.079±0.042 | -0.137±0.111# | -0.073±0.019 | -0.115±0.066 | -0.087±0.033 | -0.086±0.027 | -0.073±0.018 | -0.088±0.02 | -0.068±0.022 | -0.062±0.025 |

AP: arterial pressure; BR: baroreflex; cBR: cardiac BR; sBR: sympathetic BR; cBRS: cBR sensitivity; sBRS: sBR sensitivity; ΔAP+: positive AP variation; ΔAP-: negative AP variation; SEQ: sequence method. Data are presented as mean±standard deviation. The symbol * indicates p<0.05 versus T0 within the same type of ΔAP variation. The symbol # indicates p<0.05 versus ΔAP+ within the same experimental condition. ΔAP is intended as ΔSAP for the computation of cBRS and ΔDAP for the computation of sBRS.
